# Neurobehavioral Abnormalities Associated with Executive Dysfunction after Traumatic Brain Injury

**DOI:** 10.3389/fnbeh.2017.00195

**Published:** 2017-10-26

**Authors:** Rodger Ll. Wood, Andrew Worthington

**Affiliations:** ^1^Clinical Neuropsychology, College of Medicine, Swansea University, Swansea, United Kingdom; ^2^College of Medicine and College of Human and Health Sciences, Swansea University, Swansea, United Kingdom

**Keywords:** neurobehavioral disorder, executive dysfunction, decision making, traumatic brain injury, brain injury rehabilitation

## Abstract

**Objective**: This article will address how anomalies of executive function after traumatic brain injury (TBI) can translate into altered social behavior that has an impact on a person’s capacity to live safely and independently in the community.

**Method**: Review of literature on executive and neurobehavioral function linked to cognitive ageing in neurologically healthy populations and late neurocognitive effects of serious TBI. Information was collated from internet searches involving MEDLINE, PubMed, PyscINFO and Google Scholar as well as the authors’ own catalogs.

**Conclusions**: The conventional distinction between cognitive and emotional-behavioral sequelae of TBI is shown to be superficial in the light of increasing evidence that executive skills are critical for integrating and appraising environmental events in terms of cognitive, emotional and social significance. This is undertaken through multiple fronto-subcortical pathways within which it is possible to identify a predominantly dorsolateral network that subserves executive control of attention and cognition (so-called cold executive processes) and orbito-frontal/ventro-medial pathways that underpin the hot executive skills that drive much of behavior in daily life. TBI frequently involves disruption to both sets of executive functions but research is increasingly demonstrating the role of hot executive deficits underpinning a wide range of neurobehavioral disorders that compromise relationships, functional independence and mental capacity in daily life.

## Introduction

Executive functions represent higher level cognitive abilities that underpin many aspects of social cognition and interpersonal behavior, starting in infancy with the onset of attention control (Anderson et al., 2002) and ability to inhibit overlearned behavior (Jurado and Rosselli, 2007), progressing to the ability to infer others’ mental states (Stone et al., 1998; Zelazo and Carlson, 2012). Improvements in selective attention, working memory (WM) and problem solving occur throughout adolescence linked to late myelination and synaptogenesis in the frontal regions (Fuster, 2002; Blakemore and Choudhury, 2006). They are largely mediated by the pre-frontal cortex (Stuss, 1992; Stuss and Levine, 2002) and are therefore especially vulnerable to the mechanical forces associated with traumatic brain injury (TBI; see Bigler, 2013). For this reason, executive dysfunction lies at the heart of neurobehavioral disability (Wood, 2013) and can act as a major constraint upon an individual’s capacity for social independence. However, the way executive dysfunction and neurobehavioral disability is expressed in terms of social handicap depends upon which functions of the prefrontal system are compromised by TBI. Whilst the cognitive components of executive ability are reasonably well understood by the majority of clinical practitioners, the way in which executive dysfunction can undermine social cognition and behavioral self-regulation is often less clear. The aim this article is to review how anomalies of executive function after TBI can translate into disorders of social behavior that have an impact on a person’s capacity to live safely, and independently, in the community.

## Cognitive—Behavioral Aspects of Executive Dysfunction

The predominant cognitive deficits associated with executive dysfunction involve: (1) problems with planning, organizing, and prioritizing; (2) a lack of attentional flexibility; (3) impaired concept formation; (4) poor WM; and (5) an inability to monitor and adapt behavior consistent with changing social circumstances. These processes underpin rational thinking and for this reason are often referred to as “cold” executive functions, involving logic and reasoning. They are associated with the dorsolateral pre-frontal cortical regions (Chan et al., 2008). They are distinguished from “hot” executive functions which process emotionally salient information and comprise: (1) empathy; (2) theory of mind (ToM); (3) social judgment; and (4) emotion regulation. Hot executive functions are mediated by the ventromedial and orbito-frontal cortices (Chan et al., 2008; McDonald, 2013; Baez and Ibanez, 2014) which are implicated in the appraisal of emotional and motivational significance of events, and have both direct and indirect impact on social cognition and interpersonal behavior (Chan et al., 2008; McDonald, 2013). Indeed, our need for social interaction has led some to consider that all higher brain functions, including episodic and emotional memories, our capacity for abstract reasoning, and metacognition, have evolved solely to support interpersonal behavior and social cohesion (Frith, 2012; Shea et al., 2014). Brain activity in either hot or cold neural circuits is associated with reciprocal inhibition (Goel and Dolan, 2003) such that when one is operational the other is suppressed, which may explain why it is so difficult after TBI to exert self-control over emotional impulses.

Both hot and cold executive functions play a role in social cognition. Cold executive functions outlined above draw upon attention, memory and language and play an important role maintaining meaningful social interaction. However, these functions also rely on the ability to evaluate and interpret emotional and mental states intrinsic to social interaction, thereby involving neural substrates associated with hot executive ability. Adults with autistic spectrum disorder for example have been shown to have impairments in hot executive functions compared with controls matched on cold executive functions (Zimmerman et al., 2016). Conversely hot executive processes can predominate when people who lack mental flexibility have difficulty finding alternative ways to resolve a complex situation. Similarly the inability to monitor and update the contents of WM, characteristic of cold executive function, can undermine goal-directed behaviors, as information relevant to the intended action would not be updated and taken into account in action planning. This can result in hot executive processes driving impulsive behaviors which take precedence over pre-planned actions.

## Disorders of Impulse Control

Dickman (1990) distinguished between functional impulsivity (the ability to act without delay under pressure) and dysfunctional impulsivity, when acting without forethought leads to maladaptive responses. The latter epitomizes an imbalance between reflective and impulsive mechanisms (Strack and Deutsch, 2004) which, in the context of TBI, usually reflects an abnormality of those brain functions that mediate self-regulation. This, leads to impulsive behavior that contributes to such diverse deficits as poor tolerance, impulsive aggression, poor emotional decision-making and an amoral (pseudopsychopathic) disposition which, in combination, have implications for mental capacity. However, even though disorders of impulse control represent a frequent legacy of TBI they remain poorly understood and not always easy to recognize.

Barratt’s (1959) influential three factor model of impulsivity differentiated *motor impulsivity*, (acting without thinking); *cognitive impulsivity*, (reflecting quick decision making) and *non-planning impulsivity*, (which is largely a combination of the cognitive and motor components that represents a reactive form of behavior). Patton et al. (1995) separated the motor activation component of impulsivity (acting on the spur of the moment) from two cognitive components: *attention failure* (not focusing on the task at hand) and an *executive deficit* involving a lack of planning (not thinking carefully about options). Alternatively failure at the cognitive level may be due to inability to inhibit pre-potent responses and resist proactive interference in WM that characterized impulsiveness after TBI (Rochat et al., 2013).

More recently Whiteside and Lynam (2001) introduced the UPPS four dimensional model of impulsivity (Urgency, Perseverance, Premeditation and Sensation-seeking). *Urgency* refers to the tendency to experience and act on strong impulses, frequently under conditions of negative affect. *Perseverance* (lack of) refers to an individual’s inability to remain focused on a task that may be boring or difficult. *Premeditation* (lack of) refers to the inability to think and reflect on the consequences of an act before engaging in that act. *Sensation seeking* refers to the tendency to enjoy activities that are exciting and the willingness to try new experiences. The neural basis for each of these putative stages remains to be detailed.

It is clear from the various constructs referred to above that there may be several substrates of inhibitory control that mediate impulsive behavior, each linked to different regions of the prefrontal cortex. Bechara and Van Der Linden (2005) proposed that the ventromedial prefrontal cortex (vmPFC) and orbito-frontal cortex (OFC) are generally considered the principal regions controlling self-regulated behavior. vmPFC dysfunction influences how inhibitory control mediates decision making, such as preparing to act (Brass and von Cramon, 2002), adaptive thinking—to switch between response alternatives (Dove et al., 2000), and inhibiting inappropriate responses during strategy tasks (Shallice and Burgess, 1991). OFC dysfunction was considered by Fuster (1999) to undermine capacity for *response inhibition*, a process that normally helps maintain goal-directed behavior. Sohlberg and Mateer (2001) regard *impulse control* as an ability mediated primarily by the OFC to inhibit automatic response tendencies that usually allow flexible goal-directed behavior. They proposed that an impairment of response inhibition may result in impulsive responding, stimulus-boundedness and perseveration that can have an adverse impact on various forms of decision making. In extreme forms, utilization behavior may be exhibited, which Shallice et al. (1989) argued arose when perceptual attributes of an object automatically trigger a behavioral response in the absence of supervisory attentional control. Fuster (1999) argued that impulsive responding to random environmental stimuli arose as a consequence of distractibility, thereby side-tracking planned, goal-directed behavior, and providing a link between attention control (a cold executive function) and poor impulse control. Consistent with this hypothesis Horn et al. (2003) using fMRI showed that on a response inhibition task (Go/No-Go) impulsive adults showed greater brain activation in paralimbic areas whereas less impulsive individuals showed higher levels of activation in cortical association regions.

## Disorders of Inhibitory Control

Disorders of inhibitory control are a category of neurobehavioral deficit that includes impulsive behavior but also behavior which is not impulsive but is otherwise ill-judged or inappropriate in the broader social context in which it occurs. Thus, whilst poor inhibitory control underlies impulsive acts, many disinhibited behaviors are more accurately understood as a failure to appraise the action in context and recognize the normal social constraints on certain behaviors. The problem of disinhibition may be one of nature (the behavior is inappropriate to a specific context) or degree (the behavior is carried out to extreme levels which makes it unacceptable). Illustrations of the former include liberal use of swear words in a family setting that might be tolerated in some workplaces, and sexual innuendo that might be acceptable within an intimate relationship with a partner but would be intrusive or overly-personal in other social contexts. Research on the impact of TBI has identified social and sexual disinhibition as significant neurobehavioral factors affecting the quality of relationships (Anderson et al., 2002; Wood et al., 2005).

Physiologically, inhibition operates at different levels, the highest of which is voluntary inhibition which is centered on executive processes, although some consider inhibition to be a behavioral manifestation of several different executive processes (Bari and Robbins, 2013). Braver (2012) proposed a dual process model of inhibition whereby a *proactive* control mode reflects sustained and anticipatory maintenance of goal-relevant information and a *reactive* control mode which responds to stimulus-driven influences. De Pisapia and Braver (2006) placed these systems within the anterior cingulate (AC) and prefrontal cortex respectively though the lateral prefrontal cortex is also involved in maintaining top-down goal representations, whilst posterior and subcortical regions are engaged in task-specific processing. This allows attentional selection of goal-related information when faced with competing stimulus demands. Recent interest has focused on the role of the right inferior frontal gyrus (Chikazoe et al., 2007; Hampshire et al., 2010). Damage in this area is associated with disruption of inhibitor processes (Aron et al., 2003) whilst direct stimulation can reduce impulsive behavior (Jacobson et al., 2011). Aron et al. (2014) have argued that this area is a key part of a fronto-subcortical braking system that is normally under executive control and mediates contextually appropriate behavior. Evidence is accumulating that multiple brain regions are recruited in maintaining socially acceptable behavior, mediated largely by right hemisphere areas (Starkstein and Robinson, 1997; Garavan et al., 2006).

## Impulsive Aggression

Impulsive aggression is distinguished by a hair-trigger response following minimal provocation, usually out of proportion to the precipitating event (Barratt et al., 1997). In its verbal form it has been described by Dyer et al. (2006) as the principal aggressive trait after brain injury. It is clinically distinguishable from irritability that reflects a loosening of constraints on reactions to everyday trials and tribulations after frontal brain injury (so-called frontal irritability). Fontaine and Dodge (2006) argue that impulsive aggression arises due to automatic access to a habitual behavior, a minimal acceptability threshold and lack of executive controls. Denson et al. (2011) proposed that the threshold for eliciting anger reduces if people ruminate on a trigger event and for people with reduced executive control, resources deployed to interrupt rumination can lead to further depletion of self-control and potentially increase aggression risk. Greve et al. (2001) observed an association with post-TBI impulsive aggression and a premorbid history of irritability, impulsive or antisocial behavior. Amongst violent offenders Barratt et al. (1997) found impulsiveness *per se* was not sufficient for impulsive aggression unless poor verbal skills, in the form of developmental dyslexia, was also present. When verbal deficits interacted with low arousability thresholds impulsive aggression could be triggered in situations of conflict. This was supported by Baker and Ireland (2007) who demonstrated higher rates of dyslexia amongst offenders than non-offenders, with dyslexic traits correlating with executive difficulties and impulsiveness. In addition, dyslexic traits were also linked to more violent offences. It is also consistent with recent research on brain injury in offenders showing a link between TBI and more violent offences (Pitman et al., 2015).

Impulsive aggression has been associated with poor inhibitory control in the face of social threat at the level of the orbitofrontal and medial prefrontal cortex, resulting in an inability to control emotions generated by limbic structures such as the amygdala (Coccaro et al., 2007). This is consistent with notions that aggressive urges may be caused by hyperexcitation in a corticolimbic arousal system that includes the amygdala, AC and ventral prefrontal cortex (Keele, 2005; Brown et al., 2006).

## Disorders of Attentional Control

Attention control, also known as executive attention, refers to an individual’s capacity to choose what they pay attention to and what they ignore (Mirsky et al., 1991; Posner, 1994). Attention control therefore helps maintain a focus on task-relevant information in the presence of internal and external distraction. Thus, attention control (similar to inhibitory control) is needed to direct purposeful behavior by inhibiting the influence of irrelevant representations from gaining access to WM, within which task-related goals are retained in the face of interference by extraneous stimuli, which otherwise could disrupt the ability to maintain focus on a task and work towards achieving a goal.

Attention control is primarily mediated by prefrontal areas (including the AC cortex) that activate, regulate, and monitor how information is received and processed. It is therefore thought to be closely related to other executive functions that mediate many aspects of social cognition and human interaction (Posner and Petersen, 1990; Astle and Scerif, 2011). Posner and Petersen (1990) proposed an interactive system comprising three functional networks: alertness (maintaining awareness), orientation (information from sensory input) and executive control (resolving conflict). After TBI difficulties can arise due to inability to sustain attention (Whyte et al., 1995), attend selectively (Park et al., 1999) or depletion of overall attention resources (Azouvi et al., 2004). A disturbance that specifically affects the flexible allocation of attention towards internal representations and external information could contribute to an impression of apathy and a lack of initiative, by making the person incapable of coordinating intentions in response to changing environmental stimuli. Inattention to environmental cues can cause misperception of situations, for example failure to register nonverbal cues can undermine social interaction (McDonald and Flanagan, 2004). Low levels of attentional control are also thought to increase chances of developing anxiety because the ability to shift one’s focus away from threat information is important in processing emotions. Attentional bias can cause a person to processes emotionally negative information preferentially over emotionally positive information (Astle and Scerif, 2011).

The ability to interact in a flexible and creative way with the environment is essential for psychological health and community independence. A reduction in attention capacity and control means that people are restricted in the amount of information they can accommodate in WM. Therefore they are limited in the extent to which they can think or respond to alternatives or deal with changing environmental events. This often leads to a rigid style of thinking and behaving reflected by repetitive or stereotyped behavior. Such individuals often live according to a pre-planned schedule or time table that guides their activities during the day. If events conspire to demand changes to their schedule many individuals fail to adapt. They can therefore behave in a manner inappropriate to the situation or/and exhibit outbursts of temper because they cannot cope with the frustration and uncertainty generated by unpredictable changes to a planned schedule.

Burgess et al. (2007) hypothesized that a “supervisory attentional gateway system” flexibly allocates attention towards either internal stimuli, such as mental action plans to achieve goals or to deal with emotional states, or towards external information from the environment that demands flexibility to adjust to changing circumstances. This cognitive control mechanism, which relies mainly on the activity of the rostral prefrontal cortex (RPFC; Brodmann’s area 10), may support a wide range of situations critical to competent human behavior in everyday life, such as multitasking or remembering to carry out intended actions after a delay (Burgess et al., 2007), otherwise leading to frustration, angry outbursts, feeling of inadequacy and despondency.

## Compulsive Behavior

There is a lack of clarity about the emergence of *de novo* obsessive-compulsive behavior after TBI. Using a psychiatric frame of reference for obsessive compulsive disorder (OCD), van Reekum et al. (2000) estimated a prevalence of 6.4%, twice as common as the general population. However, Berthier et al. (1996), state that OCD has rarely been described after TBI except in individual case studies (Drummond and Gravestock, 1988; Jenike and Brandon, 1988; Donovan and Barry, 1994; Max et al., 1995) or small series lacking control groups (McKeon et al., 1984; Kant et al., 1996). Whilst no formal estimates are available from large scale controlled studies, clinical experience suggests that OCD after TBI is less common, and often different in character, to the emergence of compulsive or stereotyped behaviors that, whilst not meeting the DSM-5 criteria for OCD, nevertheless act as a constraint on adaptive social behavior. Indeed, the absence of anxiety in many cases led Wood (2001) to suggest that after TBI, novel patterns of compulsive behavior seems better classified as obsessive compulsive personality (DSM-5 301.4) than OCD (DSM-5 300.3).

In many respects, compulsive behavior after TBI appears to be an extension of a pre-accident personality characteristic, such that a person who was always methodical and organized exhibits a more concrete or rigid style of thinking leading to stereotyped behavior patterns. Unlike OCD in a psychiatric context, obsessive thoughts, urges or images which the individual tries to suppress, often associated with fears about contamination, are far less common, or intrusive, than compulsive tendencies to maintain order (what Bond, 1984, described as “organic orderliness”). Hoarding behavior, sometimes referred to as abnormal “collecting drives”, seems to be associated with an inability to decide what is useful and should be retained, as opposed to what amounts to “junk”. Anderson et al. (2005) described compulsive collecting behavior as, indiscriminate acquisition behavior and diminished discarding behavior that was blatant, repetitive and generally non-selective. Many individuals disinclination to discard objects persisted even when the “collections” led to significant negative consequences.

The development of habitual checking behavior, which develops as a novel response after TBI, is often linked to failures of WM that reflects a lack of confidence about whether or not an action (turning off the gas, electrics, etc.) has been carried out, leading to checking rituals which then develop as a habit response (see Zitterl et al., 2000). Radomsky et al. (2001) asserted that problems of attention control and information processing underpin some aspects of obsessive compulsive behavior. This view is supported by Savage et al. (2000) who suggested that memory impairment in the compulsive element of OCD is secondary to deficits of WM and executive function because patients focus on memorizing specific details but fail to have a conceptual overview, preventing details adding to a general understanding of the “big picture”.

The neurobiological basis for obsessive behavior post-TBI, is unclear. Saxena et al. (1998) proposed an orbitofronto-subcortical circuit as responsible for OCD which could easily be implicated in obsessive behaviors after TBI. The circuit involves projections from the OFC to the head of the caudate nucleus and ventral striatum, then to the mediodorsal thalamus via the internal pallidus, and finally returning from the thalamus to the OFC, a circuit which also includes connection with the basal ganglia, a system that mediates many aspects of cognition and executive function. Therefore, mechanisms of injury in TBI could have an impact on this circuit in a variety of ways. Figee et al. (2013) reviewed 37 case reports of patients with acquired OCD due to acquired brain injury and suggest that lesions in the cortico-striato-thalamic circuit, parietal and temporal cortex, cerebellum and brainstem may induce compulsivity. Post traumatic hoarding behaviors have been associated with mesial prefrontal damage. Anderson et al. (2005) investigated the occurrence of abnormal collecting behavior resulting from focal brain damage and found it could result from damage to the right mesial prefrontal region, at the level of the AC and the frontal pole.

## Decision Making

Decision-making reflects a process in which a choice is made after reflecting on the consequences of that choice. Fontaine and Dodge (2006) proposed that real-time decision making involves multiple stages of evaluation which they characterized as follows: “an individual responds to a social stimulus by perceiving stimulus cues (step 1: *encoding*), making social inferences about the stimulus and social context (step 2: *interpretation*), clarifying his or her own personal interests (step 3: *clarification of goals*), generating alternative ways to respond to the stimulus (step 4: *response access or construction*), evaluating these alternatives, considering their possible consequences, selecting the preferred response for enactment (step 5: *response decision*), and carrying out the selected behavior in response to the stimulus (step 6: *enactment*)” (p. 606). However, after TBI, many individuals exhibit poor judgment and pursue actions that lead to bad outcomes in such a manner that suggests an inability to learn from experience. Consequently, they repeat the same mistakes and lack the ability to anticipate the likely outcome of decisions. The failure to learn from repeated mistakes, against a background of normal intelligence, memory, speech, sensation, and movement, has been referred to as the *frontal paradox* (Walsh, 1985; Wood, 2001) and represents a dislocation between normal performance on measures of cognition compared to abnormal performance in the application of cognition in everyday life. Such individuals seem to exercise poor judgment when choosing friends and partners, or engage in activities that place them at some kind of risk.

The failure of decision-making that is so obvious in community activities, is often not reflected by performance on standardized clinical tests. This was one factor that led Damasio (1996) to propose a theory of decision making influenced by emotional factors, referred to as the Somatic Marker Hypothesis (SMH). The central feature of this theory is that emotion-related signals (somatic markers) assist cognitive processes in implementing decisions, especially when the outcome is ambiguous. Some somatic markers can operate below a level of conscious awareness yet bias behavioral actions, a notion that influenced Bechara’s development of the Iowa Gambling Task (Bechara et al., 2000). The IOWA examines decision-making by asking participants to make choices in circumstances that mimic real-life situations because of elements of uncertainty, reward, and punishment. This decision-making mechanism has parallels with personality traits represented by “non-planning impulsivity”, i.e., living for the moment and disregard for the future (Patton et al., 1995) or a lack of “premeditation”, the absence of thinking and reflecting on the consequences of an act before engaging in that act (Whiteside and Lynam, 2001).

Acting without thought of the consequences is considered by many to be a cardinal feature of altered personality after TBI. Impulsive decision making and poor social judgment are often accompanied by shallow affect and a lack of concern for social values, usually associated with right hemisphere prefrontal injury. The pattern of behavior after TBI has been referred to as *pseudo-psychopathy* (Blumer and Benson, 1975), or *acquired sociopathy* (Blair and Cipolotti, 2000). These terms describe the personalities of a subset of patients who lack adult tact and restraint, in association with poor social judgment and short-lived enthusiasm for ill-judged projects. Euphoric mood is sometimes accompanied by emotionally labile and erratic behavior, with low tolerance of frustration, leading to shallow irritability and impulsive aggression. Such individuals exhibit a jocular, often puerile sense of humor, making facetious comments or acting in a manner that reflects a lack of tact and restraint, usually in the form of social and/or sexual disinhibition. They exhibit a tendency to hold a favorable view of themselves that is at odds with how they are seen by others.

Disordered behavior and personality that reflects poor decision-making has been associated with injury to the vmPFC and medial orbitofrontal cortex (mOFC; Blair and Cipolotti, 2000; Bechara and Van Der Linden, 2005). Damage to medial prefrontal cortex is linked to a range of deficits in reward sensitivity, emotion based learning and decision making (Young and Koenigs, 2007; Gläscher et al., 2009). Ventromedial damage also makes people less inclined to take into account future consequences (Bechara et al., 1994) and thus incentives to action are less effective. This may help explain the increased tendency to discount future rewards after TBI, favoring short term gains in decision making (McHugh and Wood, 2008; Wood and McHugh, 2013).

## Emotional Deficits

The perception of emotionally salient information involves a complex and diverse neural system which includes the ventral striatum, specific thalamic nuclei, the amygdala, the anterior insula and regions of the prefrontal cortex (Davidson et al., 2000). At a cortical level, the ventromedial and ventrolateral prefrontal regions appear to be of particular importance for the generation of emotional responses. The ventrolateral prefrontal cortex responds to emotional information, including the induction of sad mood and the recall of personal memories and emotional material (Drevets, 2000). Functional neuroimaging has also identified dorsal regions of the AC gyrus and dorsomedial and dorsolateral prefrontal cortices in selective attention, planning and motor responses to emotional stimuli (Ochsner et al., 2002; Phillips, 2003).

Given the extensive and complex prefrontal systems mediating emotion it is unsurprising that emotion and social conduct are intimately linked (Bibby and McDonald, 2005; Henry et al., 2006; Muller et al., 2010), though it is often difficult to establish the nature of this association. For example Milders et al. (2008) failed to find any link between cognitive flexibility, emotion recognition, or ToM and proxy ratings of behavior problems 1 year post-TBI. Likewise McDonald et al. (2014) did not find evidence of a specific ToM contribution to social communication. Aboulafia-Brakha et al. (2011) were also unable to identify a specific executive process common to ToM tasks. McDonald (2013) considered the distinction between “hot” and “cold” aspects social cognition as a basis for evaluating and interpreting a social situation from the perspective of empathy. Hot social cognition was associated with emotional empathy (empathizing with the affective state of another person and actually experiencing the same emotions, but not necessarily to the same degree). Cold social cognition mediates cognitive empathy (which allows us to objectively recognize another person’s emotional state without becoming emotionally involved ourselves). Both forms of empathy are important in social interaction. However, an awareness of the context in which emotion is exhibited is also important. This can influence how we should behave in different social or environmental settings. For example, shouting emotionally charged comments whilst standing on the terrace of one’s local soccer club may be considered acceptable and even appropriate. However, the same behavior exhibited in the middle of one’s local supermarket would probably result in being arrested.

Diminished ability to experience emotion usually translates into an inability to empathize. Empathy has been described as the “*binding force*” of social cognition, allowing individuals to share experiences and understand each other’s perspectives (Eslinger, 1998). Unsurprisingly therefore, a lack of empathy can contribute to the fragility of relationships when a partner, who was previously loving and affectionate becomes, after TBI, emotionally withdrawn and indifferent. Wood and Williams (2008) explored the capacity for emotional empathy in a cohort of 89 head injured patients, 60.7% of whom recorded low levels of emotional empathy, compared to 31% of a demographically matched control group drawn from the general population. Whilst often maintaining a respectable social and emotional veneer, their behavior towards close friends and family was perceived as emotionally indifferent, with a self-indulgent attitude, often with an amoral disposition that was out of character with the individual’s pre-accident behavior.

**Figure 1:**
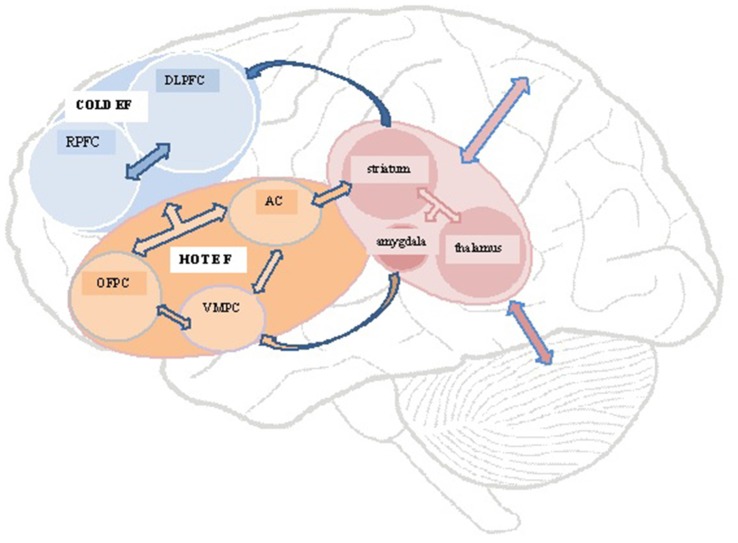
Schematic representation of structures and circuits underlying hot and cold executive functions. DLPFC, dorsolateral prefrontal cortex; RPFC, rostral prefrontal cortex; OFPC, orbitofrontal prefrontal cortex; VMPC, ventromedial prefrontal cortex; AC, anterior cingulate.

Williams and Wood (2010) found a link between a lack of emotional empathy after TBI and the presence of Alexithymia, a multifaceted personality construct comprising difficulty–identifying feelings; distinguishing between feelings and bodily sensations of emotional arousal; describing feelings to other people; constricted imaginal processes evidenced by paucity of fantasies, and a stimulus-bound, externally-oriented thinking style (Taylor et al., 1997). The impaired emotion processing and regulating capacities thought to underpin alexithymia, has led to it being conceptualized as one of several possible post TBI personality risk factors underpinning a lack of emotion recognition and responsiveness (Wood and Williams, 2007, 2008; Williams and Wood, 2010). Figure 1 presents a schematic representation of key brain structures and circuits highlighted in this review.

## Conclusion

The mechanics of TBI render especially vulnerable the fronto-temporal regions and associated subcortical structures such as the cingulate, amygdala, striatum and insula that are intimately connected to prefrontal cortex. Orbito-frontal and ventro-medial areas particularly have been implicated in a wide range of emotional and behavioral sequelae of TBI arising from disruption to hot executive functions, whilst damage to dorsolateral regions is typically associated with disturbance of cold executive processes. This distinction provides a useful means of characterizing the diverse nature of executive deficits that underlie neurobehavioral disorders and explains why traditional tests of executive abilities are often inadequate to encapsulate the range of real life problems often experienced after TBI.

## Author Contributions

The two authors are jointly responsible for the content of the article, and both contributed significantly to the submitted version. RW as first author wrote the first draft of the article; AW as second author reviewed and rewrote the article, adding significant new material which was subject to final amendment by RW and approval by AW.

## Conflict of Interest Statement

The authors declare that the research was conducted in the absence of any commercial or financial relationships that could be construed as a potential conflict of interest.
